# Pericarditis complicating COVID-19 infection: a report of two cases

**DOI:** 10.11604/pamj.2022.41.229.28816

**Published:** 2022-03-21

**Authors:** Zainab El Mir, Hammam Rasras, Falmata Laouan Brem, Zakaria Bazid, Nabila Ismaili, Noha El Ouafi

**Affiliations:** 1 Department of Cardiology, Mohammed VI University Hospital, Faculty of Medicine and Pharmacy of Oujda, Mohammed First University, Oujda, Morocco,; 2 Epidemiological Laboratory of Clinical Research and Public Health, Faculty of Medicine and Pharmacy of Oujda, Mohammed First University, Oujda, Morocco

**Keywords:** Pericarditis, myocarditis, COVID-19, pericardial effusion, case report

## Abstract

COVID-19 infection is responsible for many complications, which can lead to a high risk of mortality. Respiratory manifestations are the most encountered, while that cardiovascular complications are classified as the most severe. We report two cases of COVID-19 infection complicated by pericarditis. In the absence of other etiology of pericarditis, severe acute respiratory syndrome coronavirus 2 (SARS-CoV-2) was considered as the behind cause. The treatment in these two cases was corticosteroids with colchicine, with good outcomes. In the presence of any cardiovascular symptoms, pericarditis related to COVID-19 should be suspected, in order to act swiftly and avoid complications as well as contamination.

## Introduction

Since December 2019, the COVID-19 infection appears as a global pandemic that can attack all organs, which led to a high mortality rate around the world [1]. Many studies were carried out to describe its characteristics, complications, as well as best therapeutic approaches. Respiratory manifestations are the classic symptoms of this infection. However, other severe complications can occur, and among them come cardiac impairments, such as myocarditis, arteriovenous thrombosis, and rarely pericarditis, which can all induce cardiogenic shock that may be fatal. Their incidence is not yet established. And here is the challenge to avoid these life-threatening complications by early diagnosis and choosing the best therapeutic strategies for treating them. Here, we report two cases of pericarditis diagnosed in two COVID-19 infection-patients. We aim to describe its pathophysiology, its severity, and the different therapeutic approach suggested in this context through these cases.

## Patient and observation

**Figure 1:**
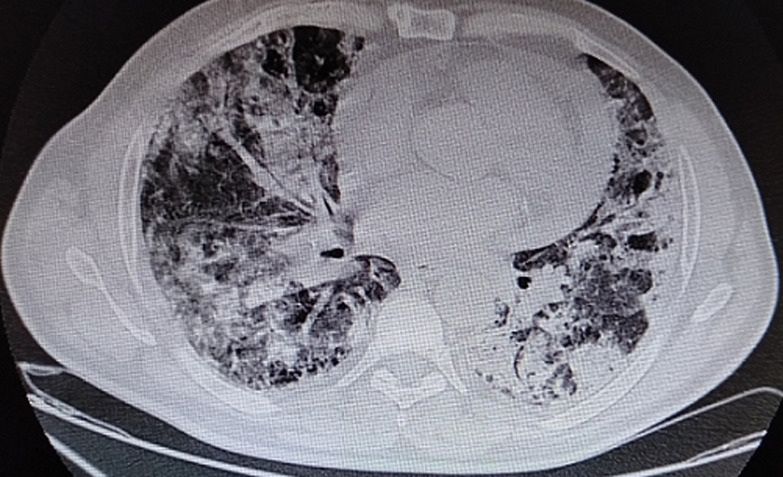
chest CT scan in axial window showed signs of COVID-19 pneumonia

**Figure 2:**
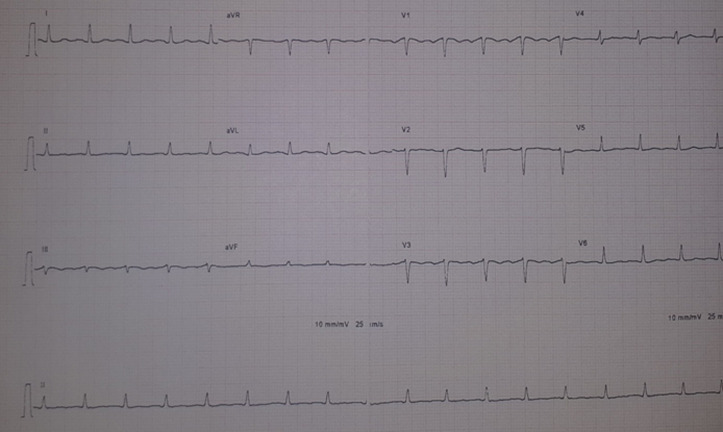
EKG showing a microvoltage and flattened T waves in circumferential

**Case 1:** this is the case of a 56-year-old man, followed for mellitus diabetes for 14 years, was admitted to for COVID-19 infection confirmed by Polymerase Chain Reaction (PCR) nasal-swab test, and thoracic Computed Tomography (CT) scan (Figure 1). On clinical examination, he was febrile (38,9°C), presented with cardiogenic shock (blood pressure (BP) at 80/40 mmHg, heart beats (HB) at 130, oliguria and mottling in both lower limbs), spontaneous SpO_2_ was 82%, HOMANS sign was negative. The electrocardiography (EKG) revealed a regular sinus rhythm with a micro voltage and flattened T waves in circumferential (Figure 2).

**Figure 3:**
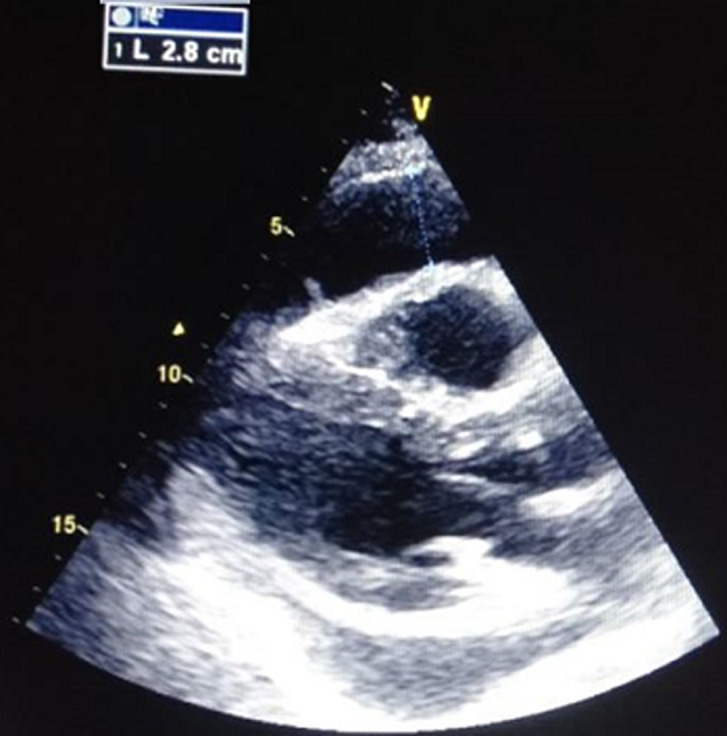
TTE showing an abundant pericardial effusion in antero-RV (right ventricle) and postero-LV (left ventricle)

In the para clinical work up, a biological inflammatory syndrome (white blood cells: 25,000/mm^3^, C-reactive protein (CRP): 315 mg/l and a procalcitonin at 4 ng/ml, fibrinogen: 8.7 g/l, lactate dehydrogenase (LDH): 1542 g/mol with ferretinemia: 2500 mg/l), was found, with lymphopenia at 300 elements/mm^3^. Ultra-sensitivity troponin (US-troponin) was high at 1868 ng/l. Trans Thoracic Echography (TTE) revealed an abundant pericardial effusion (Figure 3), without respiratory variations, global hypokinesia (with an ejection fraction at 23%), and a good function of right ventricle. The coronary angiography was without abnormalities. Therapeutic strategy chosen was to put the patient on vasoactive drugs (dobutamine and norepinephrine). For COVID-19 infection, he was treated by antibiotic coverage (ceftriaxone 2g/d, ciprofloxacin 1.5 g/d), corticosteroids, immunoglobulin, colchicine, and vitamin supplementation, with a very good outcome (on the 28^th^ day, absence of pericardial effusion, with a good left ventricular function).

**Figure 4:**
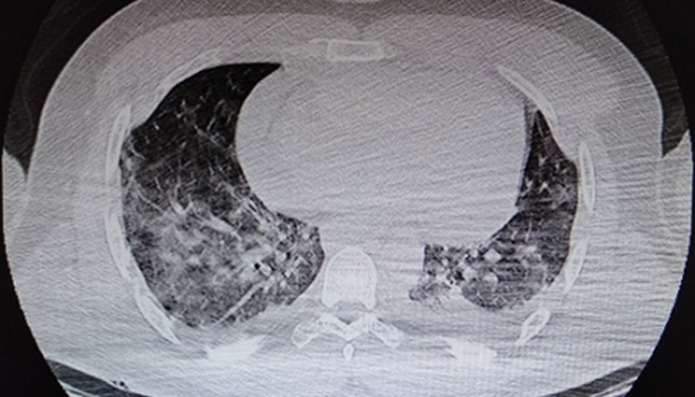
thoracic CT scan showing diffuse patchy ground-glass opacities suggesting COVID-19 pneumonia with pulmonary impairment of 50%

**Case 2:** a 56-year-old man with no significant pathological history, who presented with an acute dyspnea, after twenty days of a productive febrile cough. On clinical examination, he was hemodynamically stable (HB at 77 bpm, BP at 120/80 mmHg), spontaneous SpO_2_ was 95% in room air. According to the pandemic context, a chest CT (computed tomography) scan was performed showing bilateral pleural effusion, with impairment of 50% of pulmonary parenchyma (Figure 4), PCR for (SARS-CoV-2) on nasopharyngeal swab was positive.

**Figure 5:**
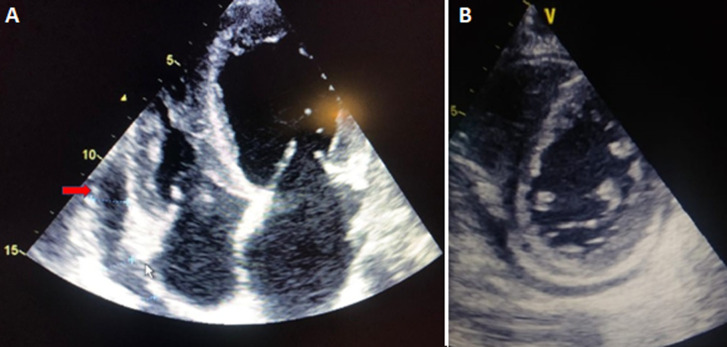
A, B) TTE showed a low pericardial effusion

In the para clinical work up, a biological inflammatory syndrome (white blood cells: 17,000/mm^3^, (CRP): 219 mg/l and a procalcitonin at 3.1 ng/ml, fibrinogen: 7.2 g/l, lactate dehydrogenase: 1102 g/mol with ferretinemia: 1781 mg/l), was found, with lymphopenia at 412 elements/mm3. US-troponin was high at 598 ng/l. The TTE showed a low pericardial effusion with biventricular dysfunction (the ventricular ejection fraction was 15%) (Figure 5). The patient was on antibiotic coverage (azithromycin with ciprofloxacin), colchicine, and corticosteroids. Fifteen days later, the patient's cardiac contractility returned normal with a systolic ejection fraction (EF) of 60% without pericardial effusion.

**Ethical approval:** given the article form, no ethical committee approval was needed (case report). However, the patients' informed permission to report their clinical data was obtained.

## Discussion

Throughout the literature, cardiac involvements complicating COVID-19 infection prove a real challenge for cardiologists and resuscitators because of the absence of predictor factors, especially in the absence of respiratory manifestations. Many studies have shown the link between coronavirus and the cardiovascular system. Its incidence is about 20% [2,3], and it can appear by myocarditis, pericarditis, heart failure and heart arrhythmia disease, in addition to the increased risk of thromboembolism [4].

Acute pericarditis is an inflammation that affects the sac around the heart [5], and in most cases, it produces pericardial effusion and can also affect the myocardium. To make its diagnosis, a transthoracic ultrasound (US) that shows the pericardial effusion is essential [5]. Transthoracic echocardiography and cardiac biomarkers must be realized for all COVID-19 infection-patients, especially in the presence of unexplained hemodynamic alteration [6].

The incidence of pericarditis complicating coronavirus is still unclear, and until now, just a few studies have been published in this domain. For its pathophysiology, two predominant mechanisms can explain this attack; firstly, SARS-CoV-2 uses the angiotensin II converting enzyme (ACE-2) as a receptor to penetrate cells, and this receptor is highly exposed in pulmonary alveolar cells in cardiomyocytes, which can cause cell damage by direct toxicity of the virus [1]. Secondly, the exaggerated inflammatory response, called (cytokine storm), which is caused by interleukins rise that induces direct toxicity on the myocyte.

In the most reported cases, pericarditis in COVID-19 infection is associated with myocarditis, left ventricular dysfunction, and cardiogenic shock, of course, without forgetting thromboembolic complications. In our patients, they had no tuberculosis contact or tumor pathology. The serologies (hepatitis B and C, Epstein-Barr virus, parvovirus 19, cytomegalovirus and picornavirus) were negative, and renal function was correct for all. The coronary artery exploration was performed for only one diabetic patient and was normal. The other had a low probability of acute coronary syndrome. According to scientific research, troponin elevation is associated with a high risk of mortality; our patients had elevated troponin levels. However, the exact significance of this elevation remains less known. It might be linked to hypoxemia, sepsis, systemic inflammatory response syndrome or an indicator of thromboembolic events (because of the state of hypercoagulability related to the COVID-19 infection), or it is probably related to viral and inflammatory heart attacks as acute myocarditis form [1].

Our patients were diagnosed with pericarditis complicating a COVID-19 infection. The inverse - pericarditis revealing a COVID-19 infection - is very rarely reported. This presentation was described in one case by Kumar *et al*. in a 66-year-old patient and another young one by Faraj *et al*. [7,8]. Although there is no strong evidence regarding the side effects of non-steroidal anti-inflammatory drugs with COVID-19 infection, their use still not recommended due to the risk of respiratory worsening. The most used treatment for pericarditis in COVID-19 infection is corticosteroids, colchicine that blocks the release of cytokines and anakinra, which is an interleukin-1 antagonist [9]. In addition to the use of corticosteroids to reduce the pathological immune response sets off by the virus, several studies have shown their effectiveness for advanced pneumonia related to the COVID-19 infection. In our series, corticosteroids were the basic treatment, in addition to colchicine outside of contraindication. Immunoglobulin was used for a myopericarditis complicated by severe left ventricle dysfunction with good progress. Unfortunately, the lack of pericardial fluid analysis has limited our report.

## Conclusion

COVID-19 is a viral infection that can affect all cardiac structures and be responsible for fatal consequences. As a cardiologist, we must think of this infection in front of any chest pain or sign of acute heart failure in the aim to act swiftly. After the improvement in their clinical condition, the two patients were gratified.
